# Impact of Multiplex PCR on Diagnosis of Bacterial and Fungal Infections and Choice of Appropriate Antimicrobial Therapy

**DOI:** 10.3390/diagnostics15081044

**Published:** 2025-04-20

**Authors:** Francesca Serapide, Rita Pallone, Angela Quirino, Nadia Marascio, Giorgio Settimo Barreca, Chiara Davoli, Rosaria Lionello, Giovanni Matera, Alessandro Russo

**Affiliations:** 1 Infectious and Tropical Disease Unit, Department of Medical and Surgical Sciences, “Magna Græcia” University of Catanzaro, 88100 Catanzaro, Italy; f.serapide@unicz.it (F.S.); pallonerita@gmail.com (R.P.); chiara.davoli93@gmail.com (C.D.); rosarialionello0@gmail.com (R.L.); 2 Unit of Clinical Microbiology, Department of Health Sciences, “Magna Græcia” University of Catanzaro, 88100 Catanzaro, Italy; quirino@unicz.it (A.Q.); nmarascio@unicz.it (N.M.); mmatera@unicz.it (G.M.)

**Keywords:** multiplex PCR, bacterial infections, fungal infections, antimicrobial therapy, antimicrobial resistance

## Abstract

Multiplex Polymerase Chain Reaction (PCR) has significantly impacted the field of infectious disease diagnostics, offering rapid and precise identification of bacterial and fungal pathogens. Unlike traditional culture methods, which may take days to yield results, multiplex PCR provides diagnostic insights within hours, enabling faster, targeted antimicrobial therapy and reducing the delay in treating critical infections like sepsis. The technique’s high sensitivity and broad pathogen coverage make it ideal for both single and polymicrobial infections, improving outcomes across respiratory, bloodstream, and bacterial/fungal infections. However, multiplex PCR is not without challenges; initial high costs and the need for specialized training can limit its adoption, especially in low-resource settings. This review discusses the clinical advantages and limitations of multiplex PCR, highlighting its influence on diagnostic accuracy, antimicrobial stewardship, and the global fight against antimicrobial resistance (AMR). Furthermore, recent innovations in multiplex PCR, such as digital PCR and portable devices, are explored as potential tools for expanding access to rapid diagnostics worldwide.

## 1. Introduction

Infectious diseases, particularly those caused by bacteria and fungi, remain a leading cause of morbidity and mortality worldwide. It was estimated that bacterial infections contributed to over 5 million deaths globally, many of which could have been mitigated with timely diagnosis and treatment [1]. Traditional culture-based diagnostics, though effective, are often slow, with results taking up to 72 h, which can delay critical interventions in severe cases like sepsis or infections in immuno-compromised patients [2].

Multiplex PCR (mPCR) has emerged as a transformative diagnostic tool, enabling the simultaneous amplification of multiple target DNA/RNA sequences from different pathogens in a single reaction [3]. Molecular tests can simultaneously detect a range of pathogens and the presence of different genes causing antibiotic resistance, reducing treatment with broad-spectrum antibiotics and turnaround time (TAT). This allows clinicians to identify bacterial and fungal pathogens within hours, significantly reducing diagnostic timelines and facilitating early, targeted antimicrobial therapy. Rapid diagnosis is particularly important in critical care settings, where timely intervention is essential for improving survival rates and reducing the length of hospital stays [4]. By minimizing the need for broad-spectrum antibiotics, mPCR also plays an important role in antimicrobial stewardship, which is crucial in managing the global crisis of antimicrobial resistance (AMR) [5].

This review examines the potential of multiplex PCR in improving diagnostic precision, optimizing antimicrobial choices, and supporting global health initiatives aimed at reducing AMR.

## 2. Multiplex PCR Technology: An Overview

Multiplex PCR technology leverages pathogen-specific primers to simultaneously detect multiple pathogens by amplifying unique DNA sequences from each target organism in a single reaction [6]. This is particularly advantageous in settings where polymicrobial infections, such as ventilator-associated pneumonia (VAP) or bloodstream infections (BSIs), are common and where rapid pathogen identification is critical for patient outcomes.

Unlike traditional culture methods, which can require up to 72 h, mPCR can provide results in 3–4 h [7]. Traditional culture is considered the gold standard to obtain the final identification of bacterial/fungal species. However, the long time, compared with mPCR, between specimen collection and definitive identification leads to increased rates of inappropriate antimicrobial therapy. The high sensitivity and specificity of mPCR, often equivalent to or exceeding single-target PCR, allow for accurate pathogen detection even when the target organism is present in low abundance (see Table 1) [8].

Culture takes 48–72 h to obtain results and has moderate sensitivity and cost efficiency. Single-target PCR provides results in 3–4 h, with high sensitivity and cost efficiency. Finally, multiple PCR detects multiple pathogens in a short time (3–4 h), but has high sensitivity and moderate cost efficiency. Digital PCR (dPCR), an emerging form of mPCR, offers even greater precision by enabling absolute quantification of DNA, reducing issues of sample variability, and enhancing sensitivity for low-abundance pathogens [9]. However, dPCR also has limitations. The fact that it starts with smaller sample volumes may have an obvious impact on the sensitivity of the technique itself, the speed of availability of results, which cannot currently compete with faster qPCR-based applications, and the cost. It must also be kept in mind that dPCR is in the early stages of its development and that it is assumed that its clinical applications will evolve toward applications more suited to healthcare needs [10]. Moreover, the development of nanoparticle-enhanced PCR and point-of-care (POC) devices holds promise for making mPCR technology accessible in low-resource or decentralized healthcare settings [11]. These POC devices are used more and more frequently, and their employment in the United States is estimated to increase by 15% over the coming years [12].

The unique ability of mPCR to test for multiple pathogens simultaneously makes it a valuable tool in clinical diagnostics, especially for complex infections where identifying the correct causative agents quickly can significantly alter patient outcomes and reduce healthcare costs. Indeed, by exploiting the absolute quantification of digital PCR, it has been possible to design assays to detect targets that have been tested only rarely, or for which there was a lack of standardized quantitative material such as GB virus and human T-cell lymphotropic virus 1. Applications also continue in the field of genetics, in the detection of hereditary diseases, and in the identification of specific bacterial pathogens (e.g., *Chlamydia trachomatis* and MRSA) [13]. However, it should be pointed out that these are emerging techniques, and therefore, efforts are also focused on trying to overcome existing limitations by enhancing the advantages.

## 3. Impact of Multiplex PCR on Diagnostic Outcomes

Multiplex PCR has been shown to outperform traditional culture methods in both sensitivity and speed, particularly in detecting fastidious organisms or pathogens and resistance patterns in patients who have already received antibiotics [14]. Studies have demonstrated that mPCR-based diagnostics can reduce time-to-diagnosis for bloodstream infections by 40%, allowing for quicker adjustments to pathogen-specific antimicrobials and improving survival rates in critical care environments [15].

For instance, in cases of bloodstream infections, mPCR has allowed clinicians to switch from broad-spectrum to targeted antibiotics within hours rather than days, as noted in a recent study which reported a 20% reduction in mortality among septic patients following rapid mPCR diagnostics [16]. Additionally, a study on respiratory infections in ICU settings revealed that mPCR reduced hospital stay duration by an average of two days, underscoring its potential in resource conservation, and improved patient outcomes [17]. These studies highlight the profound impact of mPCR on clinical decision-making, accelerating the diagnostic process and improving overall healthcare quality.

## 4. Influence of Multiplex PCR on Antimicrobial Therapy Choice

### 4.1. Antibiotic Stewardship and the Role of Multiplex PCR

Antibiotic stewardship, which aims to ensure that antibiotics are used appropriately, is essential for combating the global threat of antimicrobial resistance. Multiplex PCR’s ability to rapidly detect specific pathogens and antibiotic resistance genes enables clinicians to select narrow-spectrum antibiotics rather than relying on broad-spectrum agents [18]. This precision reduces unnecessary antibiotic exposure, thereby decreasing the likelihood of AMR development.

Incorporating mPCR into routine clinical practice supports antibiotic stewardship by providing pathogen-specific results that guide targeted antimicrobial therapy within a short timeframe. For instance, in a study on bloodstream infections, the use of mPCR led to a 30% reduction in the use of broad-spectrum antibiotics and reduced microbiological reporting time, supporting a tailored approach to therapy that minimizes the development of resistance and inappropriate empirical therapy [19].

### 4.2. Clinical Case Studies: Multiplex PCR in Antimicrobial Decision-Making

Several case studies emphasize the role of mPCR in guiding antimicrobial therapy decisions. In an ICU cohort of septic patients, mPCR enabled the early identification of pathogens, allowing for a switch to targeted antibiotics almost 48 h earlier than culture-based diagnostics [16]. This timely intervention not only improved patient outcomes but also reduced healthcare costs associated with prolonged hospital stays and unnecessary treatments. Similarly, another study [20] showed that mPCR for respiratory infections decreased the length of stay by 1–2 days, reducing antibiotic usage by 30% compared to traditional diagnostics. These findings underscore the effectiveness of mPCR in enabling rapid, informed treatment decisions that directly benefit patient care and resource allocation in healthcare settings. In summary, multiplex PCR is characterized by a faster diagnosis and a greater impact on antimicrobial resistance management, while traditional culture offers a more time-consuming but consolidated process with a smaller impact on resistance management (see Table 2).

## 5. Challenges and Limitations of Multiplex PCR

While multiplex PCR offers substantial benefits, it is not without challenges. False positives may occur due to contamination, particularly when closely related organisms share genetic similarities, which may lead to misidentification and inappropriate treatment decisions [21]. Additionally, interpreting mPCR results requires expertise to distinguish between true infections and colonization, especially in samples like blood or respiratory secretions, where pathogen presence does not always indicate an active infection [22].

The high initial costs associated with mPCR equipment and consumables present another barrier, particularly in low-resource settings where healthcare budgets are constrained. The specialized training needed for laboratory personnel to operate and interpret mPCR assays further restricts accessibility, especially in decentralized and rural healthcare settings [23]. A study by Zhang et al. [24] indicated that despite mPCR’s long-term cost savings due to faster patient recovery and reduced hospital stays, the upfront costs still pose a significant limitation (see Table 3).

Ongoing research seeks to address these limitations by developing portable and lower-cost mPCR devices. These innovations aim to expand the accessibility of mPCR technology to a broader range of healthcare facilities, thus maximizing its impact on public health and antimicrobial resistance efforts.

## 6. Applications of Multiplex PCR in Different Infection Types

### 6.1. Respiratory Infections

Respiratory infections present diagnostic challenges due to overlapping clinical presentations among bacterial, viral, and fungal pathogens. Multiplex PCR panels, such as the *FilmArray Respiratory Panel 2.1 plus*, can simultaneously detect 23 common respiratory pathogens, such as 19 viruses including SARS-CoV-2 and 4 bacteria: *B. parapertussis*, *B. pertussis*, *C. pneumoniae*, and *M. pneumoniae*. In addition, FilmArray Pneumoniae Panel plus identifies 15 typical bacteria (*Acinetobacter calcoaceticus-baumannii complex*, *Enterobacter cloacae complex*, *Escherichia coli*, *Haemophilus influenzae*, *Klebsiella aerogenes*, *Klebsiella oxytoca*, *Klebsiella pneumoniae group*, *Moraxella catarrhalis*, *Proteus* spp., *Pseudomonas aeruginosa*, *Serratia marcescens*, *Staphylococcus aureus*, *Streptococcus agalactiae*, *Streptococcus pneumoniae*, and *Streptococcus pyogenes*) and 3 atypical bacteria (*Chlamydophila pneumoniae*, *Legionella pneumophila*, and *Mycoplasma pneumoniae*); 8 viruses (Influenza B, Adenovirus, Coronavirus, Parainfluenza virus, Respiratory Syncytial virus, Human Rhinovirus/Enterovirus, Human Metapneumovirus, and Middle East Respiratory Syndrome Coronavirus (MERS-CoV)), and 7 antibiotic resistance genes [CTX-M for cephalosporin (cefepime), KPC, NDM, Oxa-48 like, VIM, IMP for carbapenemase resistance (meropenem), Mec-A/MecC, and MREJ for oxacillin resistance]. This rapid identification enables clinicians to distinguish viral from bacterial infections, reducing unnecessary antibiotic prescriptions and supporting more targeted treatments [25].

The use of mPCR provides the opportunity to overcome many challenges with improved sensitivity and turnaround time, combined with comprehensive detection abilities that support clinicians in identifying a causative agent without relying on a hypothesis-driven approach [26].

A study conducted by Feng et al. (2021) [17] demonstrated that using mPCR for respiratory infections reduced hospital stays by an average of two days and decreased antibiotic use by 25% compared to traditional diagnostics. This outcome not only benefits patient care but also decreases healthcare costs and mitigates AMR risks associated with antibiotic overuse in viral infections. In particular, several studies have evaluated the potential impact of mPCR on AMS in patients with suspected pneumonia, revealing that the majority (70–80%) of patients would be eligible for an antimicrobial change [8], including the opportunity to de-escalate in 48% and escalate in 13% whose empirical regimen did not cover the identified pathogen [27]. However, it is important to point out that studies evaluating the impact of mPCR for pneumonia on actual changes in antimicrobial therapy are limited, and more studies will be needed to demonstrate the observed changes.

During the first wave of the SARS-CoV-2 pandemic, about 30% of hospitalized COVID-19 patients were admitted to intensive care units (ICUs) for acute respiratory failure and most of them were ventilated [28]. Hospital-acquired pneumonia (HAP) and ventilator-associated pneumonia (VAP) are the most common healthcare-associated infections in ICU patients and leading causes of death [29]. Thus, empirical treatment, which may include broad-spectrum antibiotics, is frequently introduced for 48–72 h before obtaining the results of the microbiological analyses. Using standardized RT-dPCR methods to improve accuracy and reproducibility and to facilitate quantitative equivalency across different testing locations represents a step forward in the field of SARS-CoV-2 RNA load measurement. This work lays the foundation for additional studies by providing new insights into the implications of SARS-CoV-2 viral RNA load in vulnerable pediatric patients [30].

Rapid characterization of bacteria causing infections is thus pivotal in the management of severe COVID-19 patients, and thus so is the appropriate use of antibiotics [31]. According to a study by Paz et al. [32], PCR panels prevented the initiation of empirical antibiotic treatment in two-thirds of patients and led to de-escalation in more than two-thirds of those who had started empirical antibiotic treatment. The high negative predictive value of the PCR panel allowed for the diagnosis of bacterial respiratory superinfection to be ruled out. This tool represents a significant contribution to diagnostic stewardship, allowing us to avoid the unnecessary use of antibiotics.

### 6.2. Bloodstream Infections

Bloodstream infections (BSIs), including sepsis, are critical infections with high mortality rates if untreated. Traditional cultures may take days to identify causative agents, but mPCR can provide results within hours (1–1.5 h), allowing for the immediate initiation of targeted antimicrobial therapy [33]. Multiplex PCR assays, such as those targeting pathogens like *Staphylococcus aureus*, *Escherichia coli*, and *Candida* species, are particularly useful in ICU settings, where rapid pathogen identification is essential [34].

A recent study found that implementing mPCR for BSIs in ICU patients improved appropriate antimicrobial use by 30%, significantly reducing mortality rates compared to culture-based diagnostics [16]. Moreover, Frens et al. [35] (Frens, JAC 2024) highlighted the procedural improvement in rapid diagnostic testing for bloodstream infections (BSIs) by using the BioFire^®^ FilmArray^®^ BCID2 Panel (bioMérieux, Marcy-l'Étoile, France) to identify 14 Gram-negatives, 9 Gram-positives, 7 *Candida* spp., and several resistance genes, including *bla*_CTX-M_, *bla*_KPC_, *bla*_OXA-48-like_, *bla*_IMP_, *bla*_VIM_, *bla*_NDM_, *vanA*/*B*, mcr-1, CTX-M, mecA/C, and MREJ. This capability underscores mPCR’s potential in sepsis management, where early intervention is critical for patient survival.

### 6.3. Fungal and Parasitic Infections

Fungal infections pose diagnostic challenges, particularly in immunocompromised patients where traditional culture methods often fail to detect pathogenic fungi in a timely manner. Indeed, although culture-based approaches remain a mainstay of diagnosis of invasive fungal infections, they are limited by low sensitivity in patients exposed to antimycotics, and consequently, delays in diagnosis are common [36]. Furthermore, conventional biomarkers that have become crucial for the diagnosis of invasive fungal infections and invasive aspergillosis, such as 1,3-β-d-glucan (BDG) and galactomannan (GM), respectively, are negatively affected in patients receiving prophylaxis or active treatment against molds [37]. Multiplex PCR assays have shown efficacy in identifying fungi such as *Candida* and *Aspergillus*, providing results faster than culture-based methods and enabling prompt initiation of antifungal therapy [38]. The multiplex dPCR system has great potential in the diagnosis of parasitic infections, making it a promising quantitative tool in clinical practice. Blood smear examination by conventional light microscopy is the gold standard for the diagnosis of malaria. However, digital PCR (dPCR), as a method of absolute quantification detection, can be exploited as an effective tool to facilitate the diagnosis and classification of different malaria species as well as serving as a monitor in assessing the efficacy of drug treatment [39].

Multiplex PCR is particularly valuable in distinguishing between invasive infections and mere colonization in patients at high risk of fungal infections. A study published in Clinical Microbiology Reviews indicated that mPCR helped reduce the spread of invasive fungal infections in high-risk hospital settings by enabling early targeted treatment and infection control measures. Rapid diagnosis of fungal infections through mPCR minimizes the risk of disseminated infections and improves outcomes in immunocompromised populations.

## 7. Impact of Multiplex PCR on Selection of Appropriate Antimicrobial Therapy: Empiric and Definitive Treatment

The ability of multiplex PCR to rapidly identify specific pathogens plays a critical role in guiding both empiric and definitive antimicrobial therapy [40]. Early in the course of infection, particularly in severe cases such as sepsis or pneumonia, clinicians often rely on empiric therapy, typically broad-spectrum antibiotics, to cover a range of potential pathogens [41]. However, the delayed pathogen identification associated with traditional culture methods often prolongs the use of these broad-spectrum agents, which can lead to adverse patient outcomes and promot antimicrobial resistance (AMR) [42,43]. The introduction of mPCR into clinical practice enables faster pathogen identification, allowing clinicians to quickly narrow therapy based on precise microbial data, thus optimizing treatment efficacy and stewardship.

### 7.1. Empiric Therapy and the Role of Multiplex PCR

Empiric therapy is an essential early intervention in suspected bacterial and fungal infections, especially in critically ill patients, where delays in treatment can lead to rapid deterioration and increased mortality risk [44]. In the absence of definitive microbial data, empiric therapy typically includes broad-spectrum antibiotics aimed at covering a wide array of possible pathogens. However, the use of broad-spectrum antibiotics is associated with multiple risks, including toxic side effects, disruption of the patient’s microbiota, and the development of AMR [45,46]. Consequently, the goal of clinicians is to limit the duration of broad-spectrum empiric therapy by transitioning to more targeted, pathogen-specific therapy as soon as possible.

Multiplex PCR has proven highly effective in shortening this transition period by providing specific pathogen identification within hours of sample collection [47,48]. A recent study found that in septic patients, the use of mPCR led to an 80% reduction in the use of broad-spectrum empiric antibiotics within the first 24 h, as pathogen-specific results allowed for the initiation of narrow-spectrum agents [16]. This rapid narrowing of therapy minimizes unnecessary antibiotic exposure and reduces adverse effects related to broad-spectrum drugs [49]. Additionally, mPCR’s sensitivity in detecting low-abundance or fastidious organisms that may not be identified by culture further enhances its utility in informing empiric therapy decisions, particularly in patients who have already been exposed to antibiotics, which can suppress bacterial growth in cultures.

### 7.2. Definitive Therapy and Optimization with Multiplex PCR

Definitive antimicrobial therapy involves adjusting treatment based on specific pathogen identification and antimicrobial susceptibility data. With traditional culture methods, this process can be delayed by 48–72 h, during which patients continue to receive empiric therapy. The delay in tailoring therapy not only increases the risk of suboptimal treatment but also contributes to healthcare costs and worsens patient outcomes. mPCR, however, provides rapid and accurate pathogen detection, allowing for definitive, pathogen-specific therapy much sooner [25].

For example, in bloodstream infections, rapid mPCR diagnostics have allowed clinicians to initiate definitive therapy within hours, leading to significantly improved patient outcomes. Aissaoui et al. [19] reported a study in which mPCR enabled a switch to targeted antimicrobial therapy in septic patients nearly 48 h earlier than culture-based methods, resulting in a 20% reduction in mortality and shorter ICU stays. In cases of ventilator-associated pneumonia (VAP) and other polymicrobial infections, mPCR’s ability to identify multiple pathogens in a single assay is especially beneficial [50]. This rapid pathogen identification supports clinicians in targeting therapy against specific bacteria and/or fungi present, thereby optimizing treatment effectiveness and reducing the likelihood of secondary infections from unnecessary antibiotic use.

### 7.3. Multiplex PCR and Antibiotic De-Escalation

Antibiotic de-escalation—transitioning from broad-spectrum to narrow-spectrum therapy based on diagnostic results—is a key component of antibiotic stewardship, as it minimizes both toxicity and AMR risk [51]. In settings where mPCR has been incorporated, studies indicate that the rapid pathogen-specific information provided has led to high rates of successful de-escalation [52]. Feng et al. [17] found that in patients with respiratory infections, the use of mPCR reduced the duration of broad-spectrum antibiotic therapy by approximately two days, as clinicians were able to de-escalate to more specific agents. This de-escalation not only improved patient recovery times but also contributed to a 25% decrease in hospital costs by reducing complications associated with prolonged broad-spectrum antibiotic use.

Furthermore, mPCR has shown particular efficacy in de-escalation for patients with suspected fungal infections, where rapid identification of specific pathogens like *Candida* or *Aspergillus* allows for the discontinuation of broad-spectrum antifungal therapy in cases where such coverage is no longer necessary [53]. This approach not only optimizes patient outcomes by preventing unnecessary antifungal exposure but also mitigates the risk of antifungal resistance, which is a growing concern in healthcare.

### 7.4. Impact of mPCR on Antimicrobial Stewardship Programs

Antimicrobial stewardship programs (ASPs) aim to optimize antimicrobial use to improve patient outcomes, reduce AMR, and minimize adverse effects [54]. The incorporation of mPCR into ASPs has been associated with substantial improvements in these goals [55] (Virk et al., 2024). By providing rapid, accurate diagnostic information, mPCR allows ASPs to track and adjust antimicrobial usage more effectively. A study by Zhang et al. [24] indicated that hospitals implementing mPCR reported a 30% reduction in the use of broad-spectrum antibiotics and a corresponding 40% reduction in antibiotic-associated complications such as Clostridium difficile infections. This rapid adjustment to narrow-spectrum therapy based on mPCR results highlights its value as a cornerstone diagnostic tool in ASPs [56].

In addition, the data provided by mPCR can be used to inform hospital-wide antibiotic policies, ensuring that empiric antibiotic protocols align with the most common pathogens detected in specific clinical settings. This real-time data capability enhances ASPs by creating a feedback loop between diagnostic results and antibiotic usage policies, promoting a more dynamic and responsive approach to infection management.

### 7.5. Clinical Outcomes and Cost Savings Associated with Rapid Transition from Empiric to Definitive Therapy

The benefits of mPCR extend beyond immediate patient care, impacting healthcare costs and resource allocation. Studies have shown that by reducing the length of hospital stays and decreasing the incidence of antibiotic-related complications, mPCR implementation can result in significant cost savings [57]. In one study examining ICU patients with bloodstream infections, hospitals that used mPCR reduced the average length of stay by 1.5 days per patient and saved approximately USD 3000 per admission due to fewer complications and faster recoveries [17].

The reduced length of stay not only translates to cost savings but also frees up ICU resources, allowing healthcare providers to treat additional patients. Furthermore, the quicker shift from empiric to definitive therapy can lead to better infection control outcomes, as patients with identified infections can be isolated and managed more effectively, reducing transmission risks within hospital settings.

Overall, mPCR has a transformative impact on the selection of both empiric and definitive antimicrobial therapy. By providing rapid, precise pathogen identification, mPCR supports early and effective empiric therapy while facilitating a quicker shift to definitive therapy, thus enhancing patient outcomes and supporting antibiotic stewardship. Through accelerated de-escalation and reduced reliance on broad-spectrum antibiotics, mPCR not only optimizes therapy but also reduces the risk of AMR, contributing to a safer, more efficient healthcare environment.

## 8. Future Directions and Innovations in Multiplex PCR

The field of multiplex PCR is rapidly advancing, with emerging innovations aimed at increasing both the sensitivity and accessibility of this diagnostic approach. Digital PCR (dPCR) represents a next-generation enhancement of mPCR, offering increased sensitivity and the ability to quantify DNA precisely, making it ideal for low-abundance pathogen detection [58]. This technology, as noted by White & Chen (2021) [9], also reduces variability across samples, enhancing reliability in clinical diagnostics. It is a highly sensitive and accurate molecular detection technique that is widely used in biomedical applications, such as in the detection of DNA traces, rare genetic mutations, and copy number variations, achieving absolute results by exploiting minute quantities of the target gene. Precisely because of its advantages, dPCR appears to be a key tool in oncology and prenatal diagnosis in the near future [59].

Nanoparticle-based PCR assays and portable point-of-care (POC) multiplex PCR devices are being developed to address limitations in cost and accessibility [60,61,62]. Portable mPCR devices hold particular promise for use in remote or resource-limited settings, as they offer a cost-effective and rapid diagnostic tool that can be deployed outside of centralized laboratories [63]. Advances in bioinformatics and artificial intelligence are also expected to enhance result interpretation, allowing for rapid, automated analysis of complex polymicrobial infections, which could support the broader implementation of mPCR across diverse healthcare settings [64,65].

As technology continues to evolve, the integration of mPCR with global health surveillance programs may become feasible, supporting rapid pathogen identification in outbreaks and improving preparedness for future pandemics. These innovations promise to expand mPCR’s role in both routine diagnostics and global infectious disease control.

Finally, these techniques could also be used in diagnostic steps for mycobacterial infections, parasitic infections, and other neglected diseases in which standard techniques show important limits regarding sensitivity and specificity.

## 9. Conclusions

Multiplex PCR has redefined the landscape of infectious disease diagnostics, offering unparalleled speed and precision in the identification of bacterial and fungal pathogens. By enabling early and specific antimicrobial therapy, mPCR significantly improves patient outcomes, reduces healthcare costs, and supports antimicrobial stewardship efforts that are essential in combating the growing threat of antimicrobial resistance [66,67,68]. Although mPCR faces challenges, such as high initial costs and the need for specialized training, advancements in digital and nanoparticle-based PCR are addressing these barriers, making it a more accessible and versatile tool for healthcare systems globally.

The continued development and deployment of mPCR in various infection types, from respiratory to bloodstream and fungal infections, showcase its potential to streamline clinical workflows and enhance diagnostic accuracy. As mPCR technology evolves, it is poised to play an increasingly important role in personalized medicine and public health, driving advancements in both routine diagnostics and pandemic preparedness [69,70,71]. Ultimately, mPCR represents a critical component in the future of precision diagnostics, with significant implications for global health initiatives aimed at reducing the burden of infectious diseases and AMR [72,73,74,75,76].

## Figures and Tables

**Table 1:** Comparison of traditional and multiplex PCR in infection diagnosis.

Feature	Traditional Culture	Single PCR	Multiplex PCR
Time to Results	48–72 h	3–4 h	1 h
Pathogen Coverage	Limited (single)	Limited (single)	Broad (multiple pathogens)
Sensitivity and Specificity	Variable	High	High
Cost	Moderate	High	Moderate to High
Requirement for Sample Size	High	Low	Low
Labor Intensity	High	Low	Low
Ease of Implementation	Standard in labs	Limited to reference labs	Varies, specialized labs

**Table 2:** Key statistics on diagnostic delays and impacts on AMR.

Parameter	Traditional Culture	Multiplex PCR
Diagnostic Time	48–72 h	1 h
Average Time to Treatment	48+ h	<6 h
Impact on AMR (Reduction)	Moderate	High

**Table 3:** Advantages and limitations of multiplex PCR.

Advantages	Limitations
Rapid pathogen detection	Risk of false positives
High sensitivity	Potential bias to distinguish between infection/colonization/previous infection
Multiple pathogen detection	High cost of equipment and assays
Effective in polymicrobial cases	Requirement for specialized training
Reduced antibiotic misuse	Limited access in low-resource settings

## Data Availability

Not applicable.
